# Stability, structural and functional properties of a monomeric, calcium–loaded adenylate cyclase toxin, CyaA, from *Bordetella pertussis*

**DOI:** 10.1038/srep42065

**Published:** 2017-02-10

**Authors:** Sara E. Cannella, Véronique Yvette Ntsogo Enguéné, Marilyne Davi, Christian Malosse, Ana Cristina Sotomayor Pérez, Julia Chamot-Rooke, Patrice Vachette, Dominique Durand, Daniel Ladant, Alexandre Chenal

**Affiliations:** 1 Institut Pasteur, UMR CNRS 3528, Chemistry and Structural Biology Department, 75724 PARIS cedex 15, France; 2 Institut de Biologie Intégrative de la Cellule, UMR 9198, Université Paris-Sud, F-91405 ORSAY Cedex, France

## Abstract

*Bordetella pertussis*, the causative agent of whooping cough, secretes an adenylate cyclase toxin, CyaA, which invades eukaryotic cells and alters their physiology by cAMP overproduction. Calcium is an essential cofactor of CyaA, as it is the case for most members of the Repeat-in-ToXins (RTX) family. We show that the calcium-bound, monomeric form of CyaA, hCyaAm, conserves its permeabilization and haemolytic activities, even in a fully calcium-free environment. In contrast, hCyaAm requires sub-millimolar calcium in solution for cell invasion, indicating that free calcium in solution is involved in the CyaA toxin translocation process. We further report the first in solution structural characterization of hCyaAm, as deduced from SAXS, mass spectrometry and hydrodynamic studies. We show that hCyaAm adopts a compact and stable state that can transiently conserve its conformation even in a fully calcium-free environment. Our results therefore suggest that in hCyaAm, the C-terminal RTX-domain is stabilized in a high-affinity calcium-binding state by the N-terminal domains while, conversely, calcium binding to the C-terminal RTX-domain strongly stabilizes the N-terminal regions. Hence, the different regions of hCyaAm appear tightly connected, leading to stabilization effects between domains. The hysteretic behaviour of CyaA in response to calcium is likely shared by other RTX cytolysins.

The adenylate cyclase (CyaA) toxin is a key virulence factor produced by *Bordetella pertussis*, the causative agent of whooping cough^1^,^2^,^3^,^4^, and is involved in the early stages of respiratory tract colonization. CyaA toxins invade eukaryotic cells and produce supraphysiological levels of cAMP that alter cell physiology, leading to cell death. CyaA is a 1706-residue long multidomain protein. Its catalytic domain is a CaM-activated, adenylate cyclase domain (ACD) located in its 364 amino-terminal residues^5^,^6^,^7^ while the carboxy-terminal moiety is responsible for ACD translocation and the hemolytic phenotype of *B. pertussis*^8^,^9^,^10^,^11^. The translocation region (TR), spanning residues 365 to 500, is crucial for the translocation of ACD across the plasma membrane^12^,^13^ and exhibits properties similar to membrane-active peptides^14^. This region is also involved in the regulation of the pore-forming activity of CyaA^13^. The hydrophobic region (HR), spanning residues 500 to 750, contains several hydrophobic segments. The toxin is synthesized as an inactive precursor, proCyaA that is converted into the active CyaA toxin upon specific acylation of Lys 860 and Lys 983 by a dedicated acyltransferase, CyaC^15^,^16^,^17^,^18^. These lysines are located into the acylation region (AR) spanning residues 750 to 1000. These two post-translational modifications are essential for the cytotoxic activities of CyaA, *i.e.*, haemolysis and ACD translocation^15^,^17^,^19^, and also for the refolding of CyaA into a monomeric holo-state as the acylations significantly improve the recovery yield of monomers versus oligomers and multimers^20^. The C-terminal part of CyaA is the cell receptor-binding domain (RD, residues 1006 to 1706)^21^. This domain is made of glycine and aspartate-rich, calcium-binding nonapeptide tandem repeats that are characteristic of a large family of bacterial cytolysins known as RTX (Repeat-in-ToXin) toxins^22^,^23^. These motifs constitute the main Ca^2+^ binding sites of the protein^24^,^25^,^26^,^27^. The RTX motifs are intrinsically disordered in the absence of calcium^28^,^29^,^30^,^31^,^32^. RD is indeed characterized by a high content of structural disorder and a significant negative mean net charge, which is partially neutralized upon calcium binding^33^, favoring a dramatic disorder-to-order structural transition^34^. CyaA is secreted across the bacterial envelope by a dedicated type I secretion machinery^35^ made of CyaB, CyaD, and CyaE proteins^8^,^36^. In the presence of calcium, as found in the extracellular milieu that CyaA reaches upon secretion, RD folds into a stable multidomain structure^34^,^37^. Calcium-induced folding of RD is required for CyaA biological activity^24^,^25^. Once secreted, RD binds in a calcium-dependent manner to the CD11b/CD18 integrin expressed on myeloid cells, such as macrophages, dendritic cells, neutrophils, and natural killer cells^21^,^38^. These cells are the primary targets of CyaA *in vivo*^38^, although CyaA can also efficiently intoxicate a variety of cells lacking the CD11b/CD18 integrin, likely through a direct interaction with their plasma membrane^26^,^39^,^40^,^41^,^42^,^43^.

CyaA is endowed with a unique mechanism of ACD delivery into eukaryotic cells: CyaA, like MARTX proteins^44^, is able to translocate its catalytic domain directly across the plasma membrane of the target cell, from the extra-cellular milieu into the cytosol^27^,^45^,^46^,^47^,^48^,^49^,^50^,^51^. Although CyaA has been extensively studied by various cellular and molecular approaches^52^ and used in several biotechnological applications, the structural and functional states of the toxin remain poorly characterized^1^,^2^. Indeed, CyaA is a large protein made of several distinct domains and exhibits a pronounced hydrophobic character, making it prone to aggregation into multimeric complexes^24^,^25^,^46^,^53^. Until now, CyaA has mainly been extracted from *B. pertussis* with a chaotropic agent (urea) or overproduced in *E. coli* where it accumulates as inclusion bodies that also need to be solubilized with urea. After purification, CyaA is usually stored in denaturing conditions, typically in the presence of 6 M urea^24^,^46^,^54^,^55^,^56^.

We recently defined experimental conditions to refold CyaA into a monomeric and functional state starting from purified toxin stored in 6 M urea. We showed that CyaA stored in 6 M urea mainly produced multimers (U-CyaA) when refolded by dialysis, dilution or buffer exchange. Toxin refolding into a urea-free, holo-CyaA monomeric and functional state (hCyaAm), was critically dependent upon molecular confinement, the presence of calcium and post-translational acylation^20^. This procedure of monomeric toxin refolding does not prevent, however, the formation of a population of multimers of CyaA (M-CyaA).

Here, we have characterized the stability properties of the calcium-loaded monomeric and functional holo-form of CyaA, hCyaAm, and provide the first in solution structural model of this toxin obtained from SAXS data. We show that once refolded, the monomeric holo-CyaA is remarkably stable in the presence of calcium: it remains in a monomeric state for several days at room temperature and resists thermal denaturation. Importantly, the structure and functions of the monomeric toxin can also be preserved on a short time basis (a few dozen minutes) even in a milieu completely depleted of calcium. These results suggest that within hCyaAm, the RTX-containing RD domain is somehow stabilized in a high-affinity calcium-binding state by the N-terminal region (1–1000) of CyaA. Conversely, calcium binding to RD also strongly affects the folding and stability of the N-terminal region of CyaA. Our results clearly indicate that the different domains of CyaA become tightly interdependent within the monomeric hCyaAm holotoxin. Structural and hydrodynamic studies, including SAXS, SEC-TDA and AUC, revealed that hCyaAm is a compact and structured protein with an anisometric shape. The SAXS results further indicate that this structure is transiently preserved in the absence of free calcium in the milieu. Finally, we showed that the monomeric hCyaAm displayed efficient permeabilization and hemolytic activities on vesicles and erythrocytes respectively, even with the absence of calcium in the medium. Hence, the calcium ions bound to hCyaAm are sufficient to maintain the structure and the membrane permeabilizing functions of the toxin. In contrast, cAMP accumulation in cell exposed to hCyaAm was observed only in the presence of sub-millimolar free calcium concentrations (>0.1 mM) in the milieu, indicating that calcium ions are actively involved in the translocation process of the CyaA toxin catalytic domain.

## Results

### Calcium-dependent stability of the monomeric CyaA toxin

**Figure 1:**
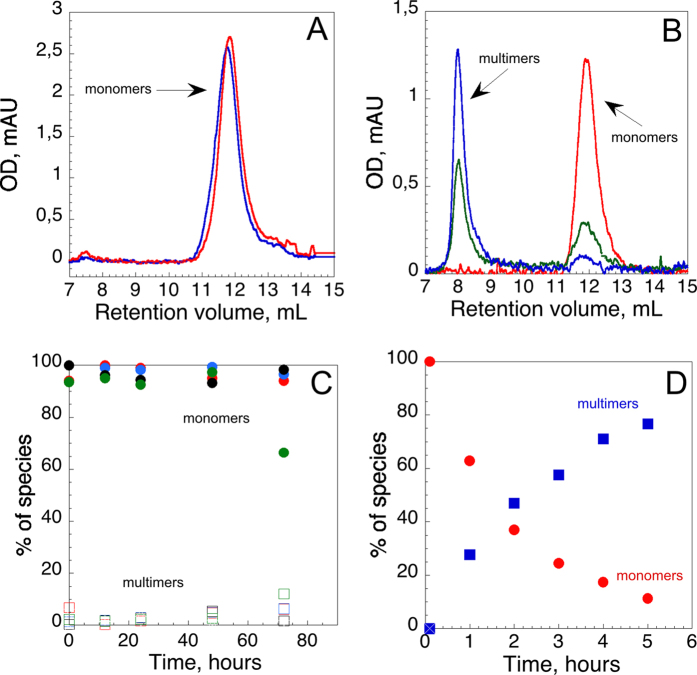
Calcium-dependent stability of hCyaAm over time. Panel (A) (buffer A complemented with 2 mM calcium) and Panel (B) (buffer A complemented with 0.2 mM EDTA): Chromatograms of hCyaAm samples at various time points showing the distribution of monomers and multimers. Samples of hCyaAm at 1 μM were loaded into the injection loop of an Äkta Pure Chromatography System (see Figure S2). At various time points, aliquots of CyaA were injected into a Superdex 200 10/300 column equilibrated with the same buffer as the hCyaAm sample loaded into the injection loop. The species of CyaA are defined according to their retention volumes, *i.e.*, multimers (8–9 mL) and monomers (11–13 mL). Panel (C) fractions of the monomers (filled circles) and multimers (open squares) were calculated by integrating the area under each peak on the chromatograms. SEC experiments were done in buffer A alone (green) or complemented with 2 (red), 0.5 (blue), 0.2 (black) mM calcium. Panel (D) hCyaAm samples were buffer exchanged on G25 column against buffer A complemented with 0.2 mM EDTA and the Superdex 200 10/300 column was equilibrated with the same buffer. Fractions of CyaA monomers (red circles) and CyaA multimers (blue squares) are shown. Buffer A contains 20 mM Hepes, 150 mM NaCl, pH 7.4. Standard deviation values: ±5%. Three independent preparations of hCyaAm were used for this experiment.

A monomeric holo-form (*i.e*., calcium-bound) of CyaA, hCyaAm, was prepared by refolding the protein in the presence of calcium using size exclusion chromatography in conditions favouring molecular confinement (*i.e*., by using small size bead media favouring excluded volume effects; see material and methods section) as previously described^20^. We first characterized the stability of this monomeric toxin at 25 °C as a function of time and in the presence of various concentrations of calcium by size-exclusion chromatography (SEC). For this purpose, the hCyaAm refolded in the presence of 2 mM calcium was first equilibrated by G25 chromatography in buffer A (20 mM Hepes, 150 mM NaCl, pH 7.4) containing various calcium concentrations (2, 0.5, or 0.2 mM CaCl_2_) or without supplemented calcium – in that latter case, the contaminating levels of free calcium in the buffers were in the range of a few μM (as determined using the calcium indicator Fura-2). The samples (1 μM of hCyaAm) were loaded into a capillary loop of an Akta pure chromatography system and injected at various time points (from a few hours to several days) onto a Superdex 200 10/300 column equilibrated with buffer A containing the same calcium concentration (*i.e*., 2, 0.5, 0.2 mM CaCl_2_ or no added calcium) as the protein samples were previously equilibrated in (Figs 1 and S2). The eluted CyaA species were named based on their respective retention volumes and oligomerization states (see SEC-TDA results in ref. 20), *i.e*., multimers (8–9 mL) and monomers (12 mL). Examples of elution profiles are shown in Fig. 1 (panels 1A and 1B and Figure S3). The proportions of each species were estimated by integrating the surface under each peak and are plotted as a function of incubation time in Fig. 1C. The data show that hCyaAm in the presence of 0.2 to 2 mM calcium is very stable at 25 °C, as the protein largely remains in a monomeric form, even after 72 hours (three days) of incubation at room temperature. In the absence of added calcium (*i.e*., with free calcium in the low μM range), the population of multimers increased after two days of incubation at room temperature. This was at the expense of monomers, indicating that hCyaAm stability is weaker in this condition. Nevertheless, even at these low calcium concentrations in solution, the monomeric toxin appears to be stable for at least 48 hours, suggesting a rather high affinity of hCyaAm for calcium.

We subsequently characterized the stability of the monomeric hCyaAm in the absence of free calcium (*i.e*., <10 nM), achieved by addition of 0.2 mM EDTA. For this, the protein was first refolded in buffer A containing 2 mM CaCl_2_ and then buffer-exchanged by chromatography on a G25 column equilibrated in buffer A containing 0.2 mM EDTA (yielding free calcium <10 nM). In these conditions, any calcium ion released by hCyaAm should be irreversibly chelated by EDTA, in contrast to the experiments performed above in the absence of added calcium (Fig. 1B, samples without calcium), where calcium ions released by hCyaAm should remain available in solution for re-binding to the protein.

**Figure 2:**
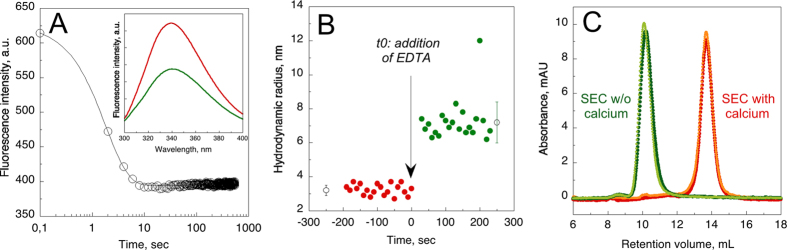
Kinetics of holo-RD unfolding upon release of calcium. Panel (A) EDTA-induced dissociation of calcium from holo-RD followed by tryptophan fluorescence spectroscopy. Two mL of 1 μM of holo-RD in buffer A +2 mM CaCl_2_ were rapidly mixed with 1 mL of buffer A +5 mM EDTA: the final concentrations of CaCl_2_ and EDTA became 1.33 mM and 1.66 mM respectively, yielding a free calcium concentration below 0.48 μM. The data show that Holo-RD is fully converted into the apo-RD state in less than 10 seconds. Panel A Inset: Fluorescence spectra of holo-RD in buffer A +2 mM CaCl_2_ (red) and 20 sec after addition of 5 mM EDTA (green). The spectra are stable on the time scale of the RD experiments. Panel (B) Light scattering analysis of the hydrodynamic radius of RD in the presence of 2 mM CaCl_2_ (red) and after addition of 5 mM EDTA (green). QELS data were collected and averaged each 10 seconds on RD (12 μM) equilibrated in buffer A +2 mM CaCl_2_. EDTA was added at time t = 0 to a final concentration of 5 mM (i.e. residual free calcium below 20 nM), and after 10 sec of mixing, QELS data were similarly collected. The average hydrodynamic radii of holo-RD and apo-RD (open circles with error bars) are 3.2 ± 0.3 and 7.2 ± 1.2 nm, respectively in agreement with our prior results^28^,^31^. Panel C: Size exclusion chromatography of RD in the presence or absence of calcium. Samples of 5 μM of Holo-RD (dark tone: in buffer A) or apo-RD (light tone: buffer A +2 mM CaCl_2_) were loaded on a Superdex 200 10/300 column equilibrated either in buffer A +2 mM CaCl_2_ (red chromatogram) or in buffer A (green chromatogram), at a flow rate is 1 mL/min. The retention volume of holo-RD (14 mL) and apo-RD (10 mL) are characteristics of the folded holo-form and the natively disordered apo-form, respectively^28^,^34^. Thus, holo-RD was fully converted to the apo-form during the chromatography time (i.e. within less than 10 minutes).

In the presence of EDTA, a conversion of hCyaAm monomers to multimers was observed with a half time of approximately 90 minutes at 25 °C (Fig. 1D). It is likely that the aggregation of the proteins resulted from the irreversible release of calcium ions from hCyaAm exposed to EDTA (Fig. 1D). Assuming that the CyaA aggregation reflects the unloading of calcium from the protein, it is noteworthy that the kinetics of calcium release from hCyaAm is dramatically slowed (t1/2 of about 90 minutes) as compared to that observed with the isolated RTX-containing C-terminal CyaA domain, RD (residues 1006–1706). Indeed, upon addition of an excess of EDTA, the calcium-loaded holo-RD protein unfolds to the apo-state in less than 10 sec as shown by kinetic measurements followed by tryptophan intrinsic fluorescence and quasi-elastic light scattering analysis (Fig. 2A and B). Calcium chelation triggers a rapid decrease (Fig. 2A; t1/2 ≈ 1.5 s) in intrinsic fluorescence, following the conversion of holo-RD to apo-RD (see emission spectra at equilibrium of both apo-RD and holo-RD in Fig. 2A inset), and elicits - within a mixing dead-time of 10 s - a large change of the hydrodynamic radius (R_H_) of the protein (Fig. 2B), from 3.2 nm, characteristic of the folded holo-RD, to a R_H_ of 7.2 nm, characteristic of the disordered apo-state^28^,^31^. These rapid and drastic changes in hydrodynamic properties were also corroborated by SEC analysis (Fig. 2C), which shows that, when chromatographed on a SEC column equilibrated in buffer lacking calcium, holo-RD was fully converted into its natively disordered apo-state within the elution time (10 min). Figure 2C further shows that the state of RD upon elution (*i*) is dependent on the buffer in which the column is equilibrated and (*ii*) is independent of the initial state of RD (apo-state or holo-state).

The remarkable stability of hCyaAm in the presence of EDTA (*i.e*., at sub-nanomolar calcium concentrations) suggests that the N-terminal moiety of CyaA (aa 1–1000) is directly interacting with the C-terminal RD domain to somehow stabilize the RTX calcium-binding sites in high affinity states exhibiting very slow dissociation rates. The tight packing of the N-terminal CyaA moiety of RD may explain the very slow kinetics of calcium release from hCyaAm as compared to that observed with the isolated RD.

Collectively, our data show that hCyaAm is remarkably stable as a monomer at 25 °C for more than three days in the presence of calcium (CaCl_2_ >0.2 mM), and for at least two days in the presence of low calcium concentrations (about 10 μM), while it aggregates into multimers in a few hours when incubated in the presence of the calcium chelator EDTA.

### Limited proteolysis of hCyaAm followed by mass spectrometry

**Figure 3:**
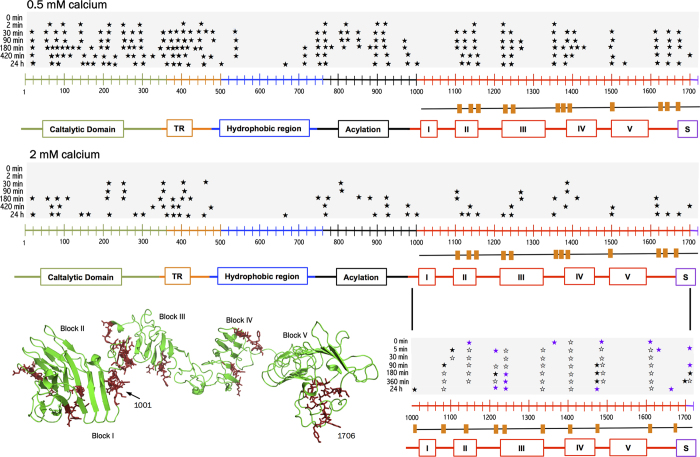
Identification of proteolytic sites in hCyaAm in the presence of 0.5 or 2 mM calcium. A batch of hCyaAm at a final concentration of 0.8 μM in buffer A complemented with 0.5 or 2 mM CaCl_2_ was incubated at 20 °C with trypsin at a final concentration of 20 nM. A sample of hCyaAm without trypsin was included as negative control. Trypsin reaction was stopped by adding AEBSF at final concentration of 200 μM and by plunging the samples into liquid nitrogen. Black stars (★) represent the proteolytic sites identified by mass spectrometry in the presence of 0.5 mM CaCl_2_ (upper panel) or in the presence of 2 mM CaCl_2_ (middle panel). The fraction of proteolysis sites over the number of amino acids in each region is 7, 11, 1.5, 5 and 3% in ACD, TR, HR, AR and RD, respectively. The same experiment has been performed on the isolated RD domain (residues 1001–1706). Black stars (★) represent proteolytic sites identified in 0.5 mM calcium, violet stars (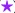) correspond to cuts identified in 2 mM calcium and open stars (☆) are proteolytic sites identified in both, 0.5 and 2 mM calcium. All proteolytic sites are listed in Tables S1 to S4. The orange boxes below the CyaA sequences correspond to the cleavage sites identified in CyaA and absent in RD while orange boxes below RD sequence correspond to proteolytic sites observed in RD only. These latter proteolytic sites, labeled in red on the SAXS-derived model of holo-RD (34), highlight the regions stabilized in the full-length toxin by the presence of other domains. Buffer A contains 20 mM Hepes, 150 mM NaCl, pH 7.4. Two independent preparations of hCyaAm were used for this experiment.

To identify the most flexible and solvent-exposed regions of CyaA, we performed limited proteolysis of hCyaAm followed by MS. Proteolysis was performed at a trypsin:CyaA ratio of 1:40 (hCyaAm at 0.8 μM) in the presence of 0.5 or 2 mM CaCl_2_ to further identify those regions whose stability is calcium-dependent. The proteolytic reaction was quenched at various time points by AEBSF and aliquots were frozen into liquid nitrogen. The procedure is illustrated in Figure S4. The proteolytic sites identified by MS at different times of incubation in the presence of 0.5 or 2 mM CaCl_2_ are shown in Fig. 3 and reported in Tables S1 to S4. Almost all proteolytic sites (>95%) identified at 2 mM CaCl_2_ were also observed at 0.5 mM CaCl_2_.

In the presence of 2 mM CaCl_2_ (Fig. 3), the most flexible and protease-accessible regions at the earliest time-points are primarily located in the catalytic domain (1–364), translocation region (365–500) and in the acylation region (750–1000), followed by RD (1006–1706), which is progressively proteolyzed over time at various sites. Finally, the hydrophobic region (500–750) is the most proteolytic-resistant domain, with only four proteolytic sites in total. This indicates that in hCyaAm, both AC and TR domains contain more flexible and solvent-exposed regions than RD and HR domains, when these are packed to each other.

In the presence of 0.5 mM CaCl_2_, MS analysis identifies roughly the same proteolytic sites, but the proteolytic cleavage is significantly faster than in the presence of 2 mM calcium. The lower calcium concentration rapidly leads to a complete degradation of the polypeptide into small fragments. This MS data indicates that although the RTX calcium binding sites are mainly located in RD, the hCyaAm stabilization induced by the increase of calcium concentration from 0.5 to 2 mM is readily observed throughout the whole CyaA protein. This suggests that calcium binding to the RTX motifs may trigger both folding and stabilization not only of the RD domain, but also of the N-terminal regions (*i.e*., residues 1 to 1000) of the toxin.

Limited proteolysis was also performed on the isolated RD domain (residues 1006–1706 of CyaA). The data indicates that the most accessible proteolytic sites were located in the regions flanking the individual RTX blocks (Fig. 3, bottom panels), in agreement with earlier studies^27^. Noteworthy, these flanking regions are not proteolyzed in the full-length toxin, suggesting that these regions are less accessible within hCyaAm and/or are stabilized by the N-terminal domains (1–1000).

### Temperature-induced unfolding of hCyaAm followed by tryptophan fluorescence

**Figure 4:**
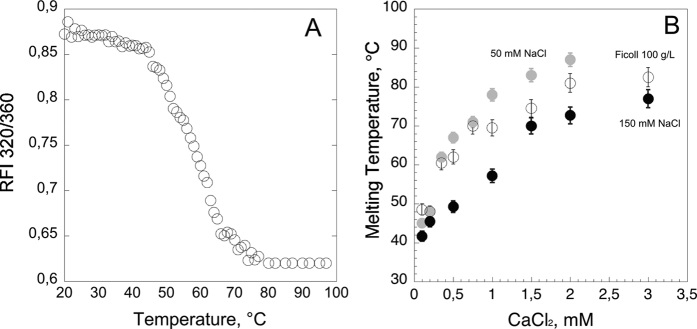
Thermal stability of hCyaAm followed by tryptophan fluorescence. Panel (A) Temperature-induced unfolding of hCyaAm at 50 nM followed by intrinsic fluorescence of tryptophan using the ratio of fluorescence emission intensities at 320 nm and 360 nm (rFI 320/360) as described in Materials and Methods. Panel (B) Effect of ionic strength and the presence of the molecular crowding agent Ficoll 100 g/L on the stability of hCyaAm as a function of calcium concentration (*i.e.*, 0, 0.2, 0.5, 1, 2 and 3 mM calcium); hCyaAm in 20 mM Hepes, 50 mM NaCl (grey circles 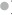), hCyaAm in 20 mM Hepes, 150 mM NaCl (buffer A, black circles ⦁) and hCyaAm in 20 mM Hepes, 150 mM NaCl, Ficoll 100 g/L (open circles ⚪). Buffer A contains 20 mM Hepes, 150 mM NaCl, pH 7.4. Error bars: S.D. Three independent preparations of hCyaAm were used for this experiment.

We then investigated the thermodynamic stability of hCyaAm at various calcium concentrations by following its temperature unfolding, as monitored by tryptophan intrinsic fluorescence spectroscopy. An example of thermal unfolding of hCyaAm is shown in Fig. 4A. The half melting temperature values, Tm, were extracted from the denaturation curves and plotted as a function of calcium concentration (Fig. 4B). In the presence of 150 mM NaCl, the Tm values increase with the calcium concentration from 43 °C (without CaCl_2_) to a plateau at around 82 °C (∆Tm = 39 °C) at CaCl_2_ concentrations higher than 3 mM. The half transition, Tm_1/2_, is reached at around 60 °C at a calcium concentration, Ca_Tm1/2_, of 1.23 mM. Similar experiments were performed at a lower ionic strength. In the presence of 50 mM NaCl, the Tm values increase with the calcium concentration from 42 °C to a plateau of around 90 °C (∆Tm = 48 °C). The half transition, Tm_1/2_, is reached around 66 °C at a calcium concentration, Ca_Tm1/2_, of 0.52 mM. This data clearly indicates that ionic strength acts as a competitor for calcium binding of hCyaAm; by decreasing the ionic strength, the Tm_1/2_ is shifted by 6 °C and the ∆Tm increased by 9 °C, while the Ca_Tm1/2_ is shifted by a factor of 2, from 1.23 to 0.52 mM CaCl_2_. These results demonstrate that calcium binding strongly stabilizes the folded conformation of hCyaAm.

We also tested the effects of molecular confinement using the crowding agent Ficoll70 in buffer A. The data show that the addition of 100 g/L of Ficoll70 stabilizes the native state of hCyaAm at the expense of its unfolded state as observed by the shift of the Tm toward higher values (Fig. 4B). We propose that the excluded volume effect due to Ficoll70 unfavours the conformational entropy of the conformers populated in the unfolded state of CyaA, thus favouring the compact and folded state of hCyaAm at higher temperatures than in the absence of molecular crowding agent. Altogether our results indicate that, in the presence of calcium, the monomeric hCyaAm is remarkably stable and resistant to thermal unfolding.

### Large unilamellar vesicle permeabilization

Membrane permeabilization of large unilamellar vesicles (LUV) by various CyaA species was assessed using the ANTS fluorescence recovery assay^12^,^14^. The permeabilization of LUV is followed by the efflux of ANTS/DPX (probe/quencher) entrapped within the vesicles, leading to fluorescence recovery of free ANTS as it dissociates from the DPX quencher, upon release into the extravesicular solution. Three different forms of CyaA were tested: (i) holo-CyaA monomers (hCyaAm); (ii) multimers of CyaA (M-CyaA) produced upon hCyaAm refolding; (iii) CyaA in 6 M urea extemporaneously diluted into buffer A in the presence of 2 mM CaCl_2_ (U-CyaA). Protein samples were tested for permeabilization of LUV made of POPC:POPG:Chol at a molar ration of 3:1:1, either in buffer A alone, in buffer A supplemented with CaCl_2_ (1, 2 or 4 mM) or 3 mM EDTA.

**Figure 5:**
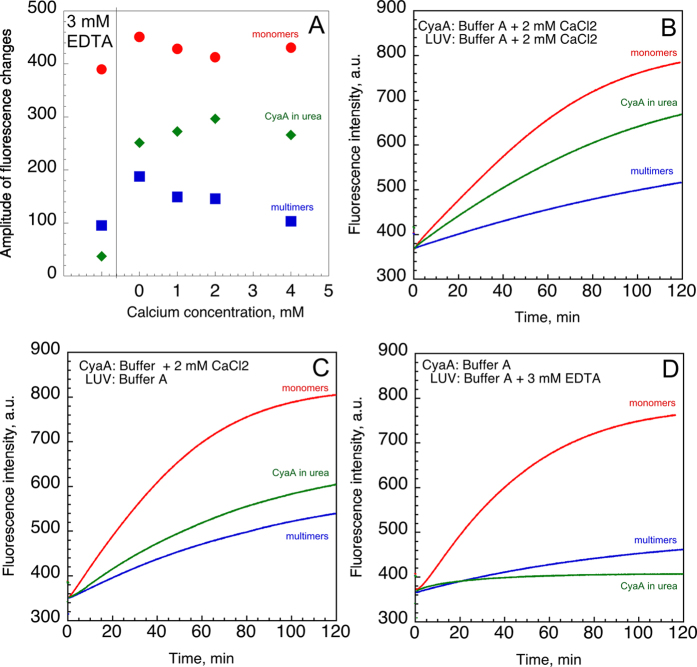
LUV permeabilization by various CyaA species. Panel (A) Amplitudes of ANTS fluorescence changes induced by monomeric holo-CyaA (hCyaAm, red circles), multimeric CyaA (M-CyaA, blue squares) and CyaA diluted from the urea stock solution (U-CyaA, green diamonds). A final concentration of 50 nM of CyaA was added to a LUV solution of 500 μM of lipids. The lipids composition of the LUV was POPC:POPG:Chol in a ratio of 3:1:1. Panel (B) Permeabilization of LUV by the different CyaA species (same color code as in panel A). Panel (C) Permeabilization of LUV (pre-incubated in buffer A) by different CyaA species pre-incubated in buffer A complemented with 2 mM CaCl_2_. Panel (D) Permeabilization of LUV (pre-incubated in buffer A and complemented with 3 mM EDTA) by the different CyaA species pre-incubated in buffer A. Buffer A contains 20 mM Hepes, 150 mM NaCl, pH 7.4. Standard deviation values in panel (A) ±30 fluorescence intensity units. Four independent preparations of CyaA were used for this set of experiments.

As shown in Fig. 5, the monomeric hCyaAm species induced membrane permeabilization at all calcium concentrations tested and also, most noticeably, in the presence of an excess of EDTA (Fig. 5A, red circles). This indicates that the calcium-loaded monomeric toxin is able to autonomously permeabilize LUV, independently of the calcium concentration in the buffer. The multimeric species of CyaA, M-CyaA (Fig. 5A blue squares and 5B-D), exhibit only a weak permeabilizing effect in the presence of calcium or EDTA, suggesting that pre-formed CyaA multimers in solution are much less efficient than monomers to permeabilize membranes. The CyaA refolded by direct dilution from a urea stock solution into a 2 mM calcium-containing medium, U-CyaA, also showed a significantly lower permeabilization efficacy (Fig. 5A; green diamonds) as compared to hCyaAm, yet, higher than M-CyaA (see Fig. 5B,C for kinetic traces).

It is noteworthy that hCyaAm does permeabilize LUV in a buffer containing an excess of 3 mM EDTA, while the two other species do not permeabilize membranes in this experimental condition (Fig. 5A and D). This result indicates that the monomeric, calcium-bound state of hCyaAm is the competent form able to efficiently permeabilize membranes in the presence of any calcium ions in the medium. This also indicates that free calcium ions in the medium/solution are not necessary for the interaction of hCyaAm with membranes and subsequent lipid bilayer permeabilization *in vitro*.

### Calcium dependency of the hemolytic and cytotoxic activities of hCyaAm

**Figure 6:**
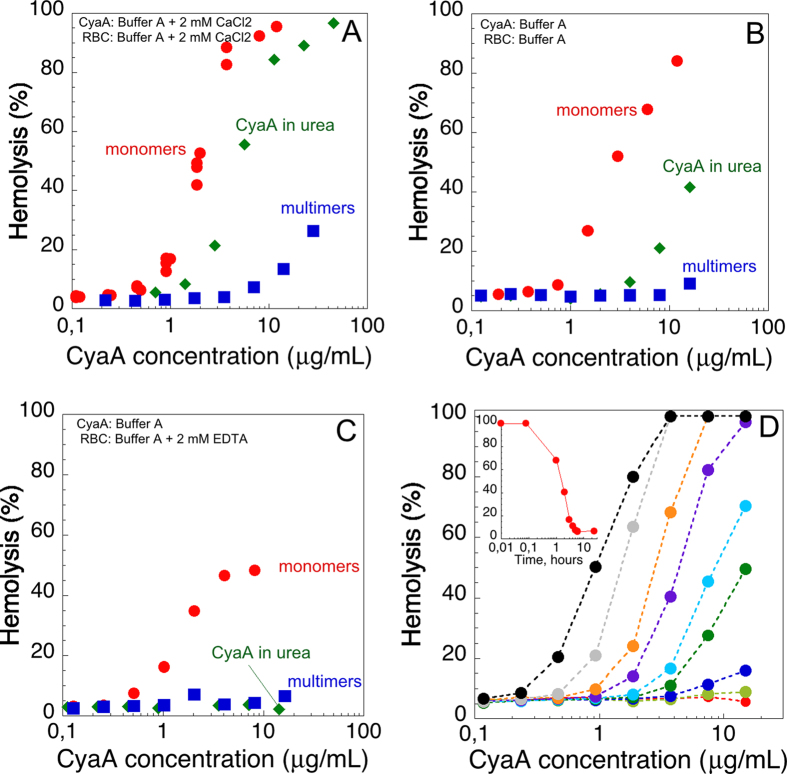
Hemolytic activity of the different CyaA species. The different CyaA samples, *i.e.*, CyaA in urea (U-CyaA, green diamonds), holo-CyaA monomers (hCyaAm, red circles), CyaA multimers (M-CyaA, blue squares), were directly diluted into erythrocyte suspensions to reach the indicated final concentrations. Panel (A) Erythrocytes were washed and resuspended in buffer A complemented with 2 mM calcium before the hemolysis assay. CyaA batches were in buffer A complemented with 2 mM calcium. Panel (B) Free calcium was removed from all CyaA samples by buffer exchange on a G25 column equilibrated with buffer A. Cells were also washed and resuspended in buffer A. Panel (C) Erythrocytes were washed in buffer A and resuspended in the presence of 2 mM EDTA. Free calcium was removed from all CyaA samples by buffer exchange on a G25 column equilibrated with buffer A. Panel (D) Excess calcium was removed from a preparation of holo-CyaA monomers by buffer exchange on a G25 column equilibrated with buffer A. The protein (2.6 μM hCyaAm) was then incubated at room temperature with an excess of 4 mM EDTA for different times, 24 hours (filled red circles), 6 hours (filled green circles), 5 hours (filled dark blue circles), 4 hours (filled dark green circles), 3 hours (filled cyan circles), 2 hours (filled violet circles), 1 hour (filled orange circles), 5 min (filled grey circles), or 0 min (plain black circles) and then directly diluted - to the final indicated concentrations - into erythrocytes, washed in Buffer A and resuspended in buffer A +4 mM EDTA. The hemolysis was recorded after an overnight incubation at 37 °C. The insert shows the % of hemolysis (measured at 20 nM final concentration of protein) as a function of time of incubation of hCyaAm with EDTA. Buffer A contains 20 mM Hepes, 150 mM NaCl, pH 7.4. Standard deviation values: ±5%. Two independent preparations of CyaA were used for this experiment.

The hemolytic activities of the different forms of CyaA were tested on erythrocytes (Fig. 6). In the presence of 2 mM calcium (Fig. 6A), hCyaAm and U-CyaA exhibited hemolytic dose-responses on red blood cells at protein concentrations higher than 1 μg/mL (5.6 nM). Multimers of CyaA, M-CyaA, also induce haemolysis but at much higher toxin concentrations, as previously observed^12^. The low activity of the M-CyaA sample may be due to an intrinsically weaker hemolytic activity of the multimeric species as compared to that of hCyaAm monomers, although we cannot exclude that it results from the presence of a small proportion of active hCyaAm monomers among fully inactive M-CyaA multimers.

In a second set of experiments, the three CyaA species, hCyaAm, M-CyaA and U-CyaA, were further desalted on G25 (against buffer A) to remove free calcium from the buffer of the samples and then incubated with erythrocytes in calcium-free buffer (Fig. 6B) or in the presence of 2 mM EDTA (Fig. 6C). In calcium-free buffer, *i.e*., in 20 mM Hepes, 150 mM NaCl, pH 7.4, with residual calcium concentrations (in the μM range), the hemolytic activity of the monomeric hCyaAm was mainly preserved while those of U-CyaA and M-CyaA were significantly reduced (Fig. 6B). When assayed on erythrocytes resuspended in buffer containing 2 mM EDTA, the hCyaAm toxin exhibited a hemolytic activity about two times weaker than that measured with 2 mM calcium, while both U-CyaA and M-CyaA species were essentially non-hemolytic (Fig. 6C). This demonstrates that the calcium-loaded, monomeric hCyaAm is hemolytic independently of the presence of any free calcium ions in the medium. Interestingly, when hCyaAm was incubated for various lengths of time in the presence of EDTA prior to the addition to the erythrocyte suspension (also in EDTA), its hemolytic activity was progressively lost (Fig. 6D), with a kinetic similar to that of the conversion of the monomeric protein to the aggregated forms seen in Fig. 1. This suggests that the calcium-loaded, monomeric hCyaAm is fully hemolytic but is progressively converted to inactive multimers upon exposure to the calcium-chelator EDTA.

**Figure 7:**
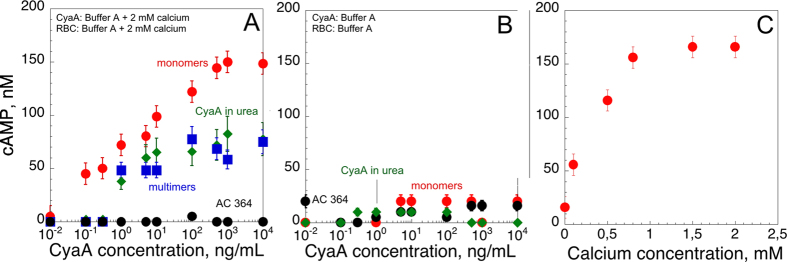
Intoxication activity of the different CyaA species. The protein samples, *i.e.*, hCyaAm (red circles), U-CyaA, (green diamonds), M-CyaA, (blue squares), AC364 (black circles) were directly diluted into erythrocyte suspensions to reach the final concentrations. CyaA in 6 M urea was buffer exchanged on a G25 equilibrated with buffer A +2 mM CaCl_2_, providing the U-CyaA sample. Panel (A) Erythrocytes were washed and resuspended in buffer A complemented with 2 mM calcium. Panel (B) All protein samples were buffer exchanged on a G25 equilibrated with buffer A to remove calcium. Erythrocytes where washed and resuspended in buffer A. Panel (C) cAMP accumulation in erythrocytes as a function of calcium concentration. Erythrocytes were extensively washed in buffer A and then supplemented with the indicated concentrations of calcium (*i.e.*, 0, 0.2, 0.5, 0,8, 1.5 and 2 mM CaCl_2_). Monomeric hCyaAm was desalted on G25 equilibrated in buffer A and diluted into the erythrocyte suspensions at a protein concentration of 2.8 nM, *i.e.*, 500 ng/mL. Buffer A contains 20 mM Hepes, 150 mM NaCl, pH 7.4. Standard deviation values: ±12 nM cAMP. Three independent preparations of CyaA were used for this experiment.

Finally, we analysed the ability of the different forms of CyaA to invade eukaryotic cells and produce intracellular cAMP (*i.e*., cytotoxic activity). Sheep erythrocytes were incubated with the different CyaA proteins, in the presence (Fig. 7A) or absence (Fig. 7B) of calcium, for 20 min, and at 37 °C. After washing, cells were lyzed and the amounts of the cAMP produced *in cellula* were determined by an ELISA immunoassay. Our results indicated that the monomeric hCyaAm toxin exhibited high cytotoxic activity as compared to the multimeric M-CyaA and U-CyaA species (Fig. 7A) in agreement with our earlier report^20^. Most importantly, the cytotoxic activity of all three CyaA forms was strictly dependent upon the presence of calcium (Fig. 7B). Figure 7C shows the calcium-dependency of the hCyaAm cytotoxic activity, which exhibits a half-maximum around 0.3–0.5 mM and reaches a plateau above 1 mM of CaCl_2_. Altogether, our results indicate that while hCyaAm is able to lyse erythrocytes in the complete absence of free calcium in the milieu, the translocation of the CyaA catalytic domain across the cells membrane is critically dependent upon the presence of sub-millimolar concentrations of free calcium in solution.

### Conformation in solution and hydrodynamics of hCyaAm

We then characterized the conformations in solution of the monomeric functional hCyaAm. To estimate the molecular dimensions of hCyaAm, we measured several hydrodynamic parameters of the protein using AUC and SEC-TDA-μV^57^. These measurements allowed us to determine the molecular mass, hydrodynamic radius, intrinsic viscosity and estimate the shape factor and the time-averaged apparent hydration of the protein (see Table S5).

**Figure 8:**
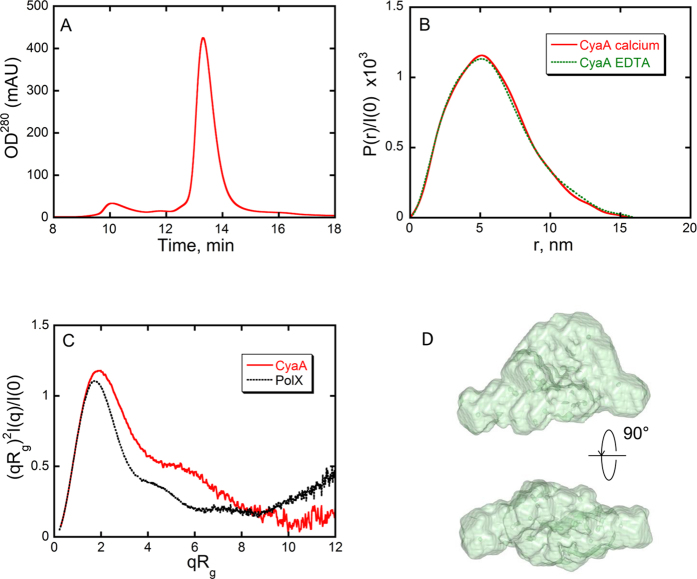
SAXS study of hCyaAm. Panel (A) UV elution profile of CyaA from the size exclusion chromatography column Bio SEC-3 equilibrated in buffer A complemented with 4 mM CaCl_2_ in-line with the SAXS measuring cell. Panel (B) distance distribution function P(r) derived from hCyaAm scattering pattern scaled to I(0). The dashed green line corresponds to the P(r) curve obtained by injecting the sample into the size exclusion chromatography column equilibrated with buffer A complemented with 2 mM EDTA. Panel (C) dimensionless Kratky plot of hCyaAm scattering pattern (red line) and PolX scattering pattern (black line, PolX is a compact, fully structured, globular protein^80^). Panel (D) Two views of the most typical DAMMIN model of CyaA. Top/bottom views are rotated by 90°. Buffer A contains 20 mM Hepes, 150 mM NaCl, pH 7.4. Two independent preparations of hCyaAm were used for this experiment.

We further analysed hCyaAm in solution by SAXS measurements carried out on the SWING beamline at the SOLEIL synchrotron radiation facility. SAXS data were recorded on protein solutions eluting from an on-line SE-HPLC column (Fig. 8A). The analysis of the hCyaAm SAXS pattern confirms that the protein eluting under the main peak is monomeric in buffer A complemented with 2 mM CaCl_2_, pH 7.4. Indeed, the values of the molecular mass derived from I(q) using two different programs and independently of the protein concentration (180 kDa and 169 kDa with SAXSMoW^58^ and Scatter^59^, respectively), are both in agreement with the sequence-based calculated value of 177 kDa.

The distance distribution function P(r) derived from I(q) suggests that hCyaAm is a compact, rather globular object, although the P(r) exhibits a moderate asymmetry, indicative of a slight shape anisometry (Fig. 8B). The values of R_g_ and D_max_ derived from P(r) are 4.53 nm and 16 nm respectively. As expected for an anisometric shape, the R_g_ value is larger than the value of 3.6 nm (ratio of 1.25) given by the empirical expression 3 (n)^1/3^ (n, number of residues) that holds true for a quasi-spherical protein. These shape parameters are confirmed by the examination of the scattering profile using a dimensionless Kratky representation (Fig. 8C) that exhibits a clear peak at qR_g_ = 1.9 and (qR_g_)^2^I(q)/I(0) = 1.18, close to the canonical values (√3, 3exp(−1)) corresponding to the Guinier law, followed by a marked decrease close to zero which describes compact and globular proteins (the scattering curve of such a globular protein, PolX, is plotted in Fig. 8C for comparison purpose). We can therefore reasonably conclude that CyaA adopts an essentially globular conformation in solution. The peak at qR_g_ = 1.9 is slightly shifted as compared to the qR_g_ value (√3 = 1.73) expected for a quasi-spherical protein. This is associated with a slight anisometry of hCyaAm that is in agreement with both the distance distribution function (Fig. 8B) and the viscosity increment (Table S5). The maximal extension D_max_ of hCyaAm is very close to that of the holo form of the receptor-binding domain (RD; 15.5 nm) reported in a previous study^34^. It is thus conceivable that both the catalytic domain and the hydrophobic region fold over RD.

We determined the shape of CyaA by using the *ab initio* modelling program DAMMIN^60^. One hundred runs yielded as many models that were superimposed and compared using the DAMAVER suite^61^. The average NSD was about 0.80, indicative of a great similarity between models. The most typical model is shown in Fig. 8D. The structural model indicates that hCyaAm is a folded, compact, anisometric and multidomain protein in solution. This model was used to calculate hydrodynamic dimensions (listed in Table S5) that were compared to those inferred from SEC-TDA-μV and AUC. Similar values are obtained for hydrodynamic parameters using both SAXS and SEC-TDA-μV.

We also analysed hCyaAm by SAXS after chromatography on a SEC column, which was equilibrated with buffer A containing 2 mM EDTA, instead of 2 mM CaCl_2_. The distance distribution function is similar to the P(r) obtained in calcium-containing buffer (Fig. 8B), indicating that the calcium-loaded structure of hCyaAm is preserved during the time of the SE-chromatography experiment, *i.e*., 20 min, in agreement with the distribution of CyaA species presented in Fig. 1D. The molecular shape of hCyaAm in the presence of EDTA is therefore similar to that found in the presence of calcium. Taken together, our results in the presence of EDTA indicate that once hCyaAm is transferred to a calcium-free environment, it transiently preserves its monomeric and native 3D structure as well as its pore-forming and hemolytic activities, while its ACD translocation ability is lost.

## Discussion

Calcium is an essential cofactor of the CyaA toxin, as it is for most other members of RTX toxin family^22^,^23^,^62^,^63^,^64^. Calcium binds to RTX repeats and is critical for the different toxin activities, including secretion, binding to the cell receptor and/or to the membrane of target cell, its pore-forming and hemolytic activities and finally for the delivery of its catalytic domain across the plasma membrane. Yet, how calcium can modulate these different properties has thus far remained unclear. Earlier work showed that calcium ions are required for the proper folding of the C-terminal RD domain^26^, which is intrinsically disordered in its apo-form and folds into a stable and compact structure upon calcium binding to the RTX nonapeptide repeats^27^,^28^,^31^,^34^. We proposed that this calcium induced folding could be directly involved in the secretion process through the type I secretion machinery^28^,^34^ and this was also recently confirmed by Bumba *et al*.^37^. More recently we found that calcium, as well as the CyaC-mediated site-specific acylation of CyaA, are essential for the correct and efficient folding of full-length toxin into a monomeric form that appears to be the functional, active species displaying both pore-forming/hemolytic activities and cytotoxic properties, *i.e*., ability to induce cAMP accumulation in target cell cytosol^20^. Interestingly, we have shown that molecular confinement during the refolding process was also critical to favour CyaA folding into the monomeric state and to prevent aggregation of the polypeptides into non-(or poorly) functional oligomeric states due to intermolecular hydrophobic forces.

Herein, we characterized the structural and stability properties of the monomeric calcium-loaded (holo) form of CyaA hereafter designated hCyaAm. We showed that hCyaAm is remarkably stable when maintained in the presence of calcium. It shows no sign of aggregation for several days at room temperature when calcium is present at concentrations higher than 0.2 mM. Even at low micromolar CaCl_2_ concentrations (5–10 μM; when no calcium is added to the medium), hCyaAm is stable for at least two days before starting to oligomerize. Furthermore, calcium was shown to strongly enhance both its thermal stability with Tm values higher than 70 °C at mM calcium concentrations, and its resistance towards proteolytic digestion (see below).

More strikingly, in a medium fully depleted from free-calcium (i.e. <10 nM), we found that hCyaAm could transiently maintain its monomeric and functional state for up to an hour (at room temperature), and was able to efficiently permeabilize lipid bilayers and lyse erythrocytes. This transient stability is most remarkable when compared to that of the isolated RD domain (residues 1006–1706 of CyaA). Holo-RD fully unfolds (as monitored by fluorescence spectroscopy and DLS) is less than 10 sec (about 2 s) upon calcium chelation by EDTA (Fig. 2), while the half-time for hCyaAm aggregation in EDTA is at least 1000 times higher (about 60–90 min) at room temperature (Fig. 1D). These results indicate that calcium binding to the isolated RD domain is a highly dynamic process. Consequently, chelation of free calcium with EDTA results in a rapid dissociation of calcium from RD and unfolding of the protein to its intrinsically disordered apo-state^28^,^31^,^34^. In contrast, the release of calcium from hCyaAm appears to be dramatically slowed, suggesting that the RTX calcium binding motifs are somehow stabilized in high affinity states within the structure of full-length hCyaAm. We propose (see Figure S5) that in folded hCyaAm, the N-terminal segments of the CyaA polypeptide, encompassing residues 1 to 1000, may be partly folded or wrapped around the RD domain, thus constraining its structure and stabilizing the RTX calcium binding motifs in a high affinity state. The calcium ions, loaded onto the RTX motifs of RD within the folded monomeric hCyaAm, would thus be buried into the structure and would experience very slow dissociation rates as compared to those of the more solvent-exposed RTX motifs in the isolated RD domain. Such interdomain interactions within hCyaAm may explain the calcium-dependent hysteretic behaviour of the toxin.

This model is corroborated by experiments of limited proteolysis followed by mass spectrometry showing that the flanking regions of RTX blocks are more accessible in isolated RD than in full-length monomeric CyaA toxin (Fig. 3). This data thus confirms that within the native, monomeric hCyaAm, RD is protected and stabilized by the N-terminal polypeptidic region. Interestingly, the limited proteolysis experiments also show that the N-terminal domains of CyaA are similarly protected against proteolytic degradation in the presence of high calcium concentrations. This indicates that the calcium-induced folding of RD^31^ directly contributes to the stabilization of the N-terminal domains of CyaA. Altogether, this data suggests a tight and concerted coupling between the folding of the C-terminal RD domain, triggered by calcium-binding, and that of the N-terminal regions of CyaA. It is interesting to note that co-stabilization effects between the C-terminal RTX domain and N-terminal region have also been observed in the *E. coli* α-hemolysin^65^,^66^.

This model is further supported by SAXS structural studies revealing that hCyaAm adopts a compact, mostly structured and roughly triangular shape in the presence of calcium. We note that the maximal extension Dmax of hCyaAm is very close to that of holo-RD (15.5 nm) reported in a previous study^34^. We attempted to localize RD within the DAMMIN envelope of hCyaAm. Using Supcomb, we superimposed the most typical pseudo-atomic model obtained for RD over the ten best-ranking DAMMIN models when ordered by increasing average NSD value. All results were very similar to the one shown in Figure S5 in which RD is seen occupying the center of the most typical model of hCyaAm with its two spatial extremities close to the two most distant vertices of the triangular shape. The localization supports the view that RD acts as a structuring agent for full-length protein, the N-terminal domains being arranged around the central spine with no apparent constraints preventing numerous and tight interactions with RD within folded hCyaAm.

Based on the ensemble of data reported here, we further propose that the calcium-loaded, folded RD domain acts as a scaffold to favour the refolding of the N-terminal domains of CyaA toward a monomeric state. This directional refolding from the C- to the N-terminus of CyaA might occur concomitantly with the vectorial secretion of the toxin through the type 1 secretion system. The N-terminal domains (1–1006 aa) of hCyaAm could establish numerous interactions with holo-RD that could explain (i) the calcium-dependent hysteresis of RD within hCyaAm as compared to the fast calcium dissociation from the isolated holo-RD domain (Figs 1 and 2) and (ii) the mutual stabilization of CyaA domains (Fig. 3). Point (i) is supported by the remarkable similarity of the SAXS pattern recorded on the hCyaAm transiently exposed to EDTA (during the on-line size-exclusion chromatography preceding SAXS data collection) to that recorded in the presence of 4 mM calcium. This indicates that the overall molecular shape of hCyaAm was essentially identical in the presence and in the absence of free calcium in solution. Altogether, these results establish that the monomeric holo-CyaA protein adopts a stable state that transiently retains its conformation even in a calcium-less environment.

These structural insights fully correlate with the functional assays of the toxin. Our results indicate that the pore-forming and hemolytic activities of hCyaAm are essentially independent of the presence of free calcium in the medium. The monomeric hCyaAm toxin thus appears as the true functional form of the toxin, able to interact with the target cell membrane and adopt a membrane-inserted state in a calcium-independent manner. It is generally assumed that the oligomerization of CyaA in the membrane is required to produce the pore-forming species^48^,^67^. Our current data suggests that this oligomerization step, if any, should also be independent of free calcium in solution. Our data also supports the view that, at variance with earlier proposals^48^, the oligomeric species that form in solution may not actually be biologically relevant.

In contrast to the membrane permeabilization and hemolytic activities, the translocation of the catalytic domain across the target cell membrane, as monitored by the intracellular cAMP accumulation, turned out to be strictly dependent on calcium, in agreement with earlier reports^40^,^68^. This indicates that, after the proper interaction and insertion of CyaA into membrane, the transport of the catalytic domain across the lipid bilayer is dependent on the presence of calcium at millimolar concentrations. It is possible that an influx of calcium is needed to favor the passage of ACD across the lipid bilayer. This model would be reminiscent of many protein transporters in which protein movements across the bilayer are driven by selective binding and transport of ions following their electrochemical gradients. Interestingly, it was shown that CyaA translocation could indeed trigger a transient rise in intracellular calcium^51^,^69^,^70^. Alternatively, high calcium concentrations may be needed to stabilize the RD domain once hCyaAm is inserted into the cell membrane. It is likely that after the initial membrane binding event, the hCyaAm protein undergoes significant conformational changes leading to a membrane-inserted state competent to translocate its catalytic domain across the plasma membrane into the cytosol. During such conformational changes, the RD domain may become fully exposed to the solvent at the surface of the membrane, and free mM calcium in solution could then be needed to maintain RD in its holo-state, as is the case for the isolated RD protein^28^,^31^. However, whether holo-RD directly contributes to the translocation process of ACD across the membrane or is involved in a still unknown activity on the plasma membrane surface remains to be established and further work will be needed to clarify these issues.

The present study highlights the remarkable stability of the calcium-bound, monomeric hCyaAm that can transiently conserve its 3D structure and pore-forming activity even when transferred to a calcium-free environment. The calcium-dependent hysteresis of hCyaAm also offers a rational explanation for some striking observations from earlier works on the characterization of the CyaA calcium binding properties^26^,^71^. It has been reported that urea stock solution of CyaA exposed to calcium or desalted by gel filtration in the presence of calcium could retain some residual membrane binding capability and hemolytic activity when tested in the presence of an excess of EGTA. We further found that few (3–5) calcium ions remained bound to the calcium-loaded protein even after size-exclusion chromatography (SEC) performed in the presence of EGTA^26^. This led us to postulate that several calcium-binding sites of high affinity exist in CyaA. From our present and other recent studies, we can now reinterpret these earlier results as follows: when CyaA was refolded by gel filtration in the presence of calcium, only a small fraction (around 10%) of the protein actually refolded into the monomeric hCyaAm form, but this fraction could then retain all of its bound calcium ions (about 30 to 40) during the SEC performed in EGTA. Altogether, this would result in a mean value of 3–5 tightly bound calcium ions per CyaA protein, as observed^26^.

The calcium-dependent hysteresis of hCyaAm is likely to be a common characteristic of all RTX toxins. In pioneering studies on the role of calcium in the structure and function of the *E. coli* α-hemolysin HlyA, Boehm *et al*.^72^ and Döbereiner *et al*.^73^ observed that the hemolytic activity of culture supernatants of *E. coli* grown in calcium-free broth was calcium dependent, while that of culture supernatants obtained from cells grown in broth supplemented with calcium was calcium independent. The calcium independent cytolytic activity of the calcium-loaded hemolysin is indicative of a similar stability of the holo HlyA protein.

In summary, we report here the first in solution 3D structural characterization of the monomeric, calcium-bound active form of full-length CyaA toxin as deduced from SAXS analysis and hydrodynamic studies. We show that hCyaAm adopts a compact and highly stable state that can transiently conserve its conformation and its pore-forming and hemolytic activities in a calcium-free environment while the ACD translocation process requires free mM calcium in solution. This species likely represents the toxin that is actively secreted by *Bordetella pertussis* through the T1SS pathway and that is competent to invade eukaryotic target cells. The remarkable properties of the calcium-bound, monomeric hCyaAm toxin in response to calcium depletion are likely shared by other RTX cytolysins.

## Methods

Production and Purification of CyaA, refolding of CyaA into holo-CyaA monomers (hCyaAm) were performed as described previously (ref. 20 and in a patent describing the production of the monomeric CyaA toxin EP 14 30 6162). Buffer A contains 20 mM Hepes, 150 mM NaCl, pH 7.4. Supplementary Figure S1 illustrates the production of holo-CyaA monomers (hCyaAm) and their separation from multimers (M-CyaA) on a Superdex200 pg column (GE Healthcare). Similar experiments were performed on BioSEC5 21.2 300 mm (Agilent) with a 5 mL G25-SF (GE Healthcare) as guard column. The production of monomers and the ratio of monomer vs multimer species are reproducible on both Superdex200 pg (n = 9) and BioSEC5 21.2 300 mm (n = 3) columns. The U-CyaA samples are prepared by buffer exchange of CyaA (protein stock solution in 6 M urea) on a G25 equilibrated with buffer A complemented with 2 mM CaCl_2_. The RTX domain (RD, residues 1006–1706 of CyaA) protein was produced and purified as previously described^28^,^31^. Calcium contaminations in buffers were measured with fluorescent indicator fura-2 (ThermoFisher Scientific) and/or calculated with the Maxchelator program (http://maxchelator.stanford.edu/CaEGTA-TS.htm)^74^.

### Analytical ultracentrifugation (AUC)

Sedimentation velocity experiments were performed on a Beckman XL-A analytical ultracentrifuge (Beckman Coulter) in an AN60-Ti rotor at 25 °C. The samples were filtrated on 0.2 μm filters before experiments. Detection of the protein concentration as a function of radial position and time was performed by optical density measurements at a wavelength of 280 nm. CyaA samples were loaded in a 1.2 mm-thick two channels epoxy centerpiece and spun at 20,000 rpm. Data were analysed with the SEDFIT software using a continuous size distribution c(s) model as described in ref. 28.

### CyaA stability over time followed by SEC

Time stability experiments were carried out at 25 °C by monitoring the distribution of protein species in the elution profiles at various calcium concentrations over time using size exclusion chromatography. Briefly, CyaA monomers at a final concentration of 1 μM in buffer A in the presence of various concentrations of calcium (0, 0.1, 0.2, 0.5 and 2 mM CaCl_2_) were loaded into the injection loop of an Akta Pure chromatography system (GE Healthcare). At selected time points, fractions of samples were loaded onto a Superdex 200 10/300 GL to fractionate the protein species and to quantify the proportions of monomers and multimers.

CyaA monomers at a final concentration of 1 μM in buffer A in the presence of various concentrations of calcium were prepared by desalting stock solution of hCyaAm in the presence of 2 mM CaCl_2_ on 5 mL HiTrap Desalting columns made of Sephadex G-25 Superfine and run on AKTA pure chromatography system. According to the provider (GE Healthcare, cf data file 28–9137–87 AA http://www.gelifesciences.co.jp/catalog/pdf/28913787aa.pdf), after desalting, the contamination of the sample by the initial buffer should be around 0.2%. As the initial calcium concentration in CyaA samples is 2 mM, the calcium carry-over in protein sample after Sephadex G-25 chromatography should be lower than 10 μM (i.e. <0.5% of 2 mM). Assuming that Hepes buffer contains approximately 3–10 μM contaminating CaCl_2_, the final concentration of calcium may differ from the theoretical value by less than 20 μM. For instance, for buffer exchange in a final 0.2 mM CaCl_2_ solution, the calcium concentration will range from 0.2 to 0.22 mM CaCl_2_ (*i.e.*, 200 μM + maximum of 10 μM of contaminating calcium in hepes buffer + maximum of 10 μM of contaminating calcium in carry-over on G25). In the absence of added calcium in the final buffer, the calcium concentration in the CyaA sample should range from 10 to 20 μM CaCl_2_. This is below the quantity of calcium required to fully saturate CyaA (assuming 35–40 calcium ions per protein) at 1 μM, *i.e.*, 35–40 μM CaCl_2_. In all cases, the differences between the targeted calcium concentrations and the exact values can be neglected.

### Calcium and EDTA-induced hydrodynamic changes of RD

Size exclusion chromatography of RD was performed on a Superdex 200 10/300 column equilibrated in buffer A only (20 mM Hepes, 150 mM NaCl, pH 7.4) and in buffer A complemented with 2 mM calcium. Samples of apo-RD and holo-RD at 5 μM were loaded into the injection loop of an Äkta Pure Chromatography System. The species of RD are monomeric in both the apo-state (10 mL) and the holo-state (14 mL). The dramatic change of retention volume is due to the change of shape and hydration, i.e., to the calcium-dependent disorder-to-order transition of RD (see ref. 28,34 for details). The flow rate is 1 mL/min, indicating that, for instance, a holo-RD sample is fully converted to apo-RD into less than 10 minutes (retention volume of the dark green trace at 10 mL in Fig. 2C) and that apo-RD is fully converted into holo-RD in less than 15 minutes (retention volume of the dark red trace at 14 mL in Fig. 2C).

EDTA-induced dissociation of calcium from holo-RD was followed by tryptophan fluorescence spectroscopy. Two mL of 1 μM of holo-RD in 20 mM Hepes, 150 mM NaCl, 2 mM CaCl_2_ were equilibrated in a QS cell and then rapidly mixed with 1 mL of 20 mM Hepes, 150 mM NaCl, 5 mM EDTA: the final concentrations of CaCl_2_ and EDTA became 1.33 mM and 1.66 mM respectively, yielding a free calcium concentration below 0.48 μM. The sample was continuously excited at 280 nm, fluorescence emission was recorded at 340 nm, and bandwidths were fixed at 5 nm for both excitation and emission. The data show that Holo-RD is fully converted into the apo-RD state in less than 10 seconds (approx. k_obs_: 0.54, *i.e.*, with a half-transition of about 1.3 sec.). Tryptophan fluorescence spectra at equilibrium of holo-RD in 20 mM Hepes, 150 mM NaCl, 2 mM CaCl_2_ and after addition of 5 mM EDTA were acquired with an excitation at 280 nm and fluorescence emission spectra were recorded from 300 to 400 nm, with bandwidths fixed at 5 nm for both excitation and emission. The spectra are stable on the time scale of the RD experiments.

Light scattering analysis of the hydrodynamic radius of RD was performed in the presence of 2 mM CaCl_2_ and after addition of 5 mM EDTA. QELS data were collected (and averaged each 10 seconds) on a NanoZS instrument (Malvern) as described^31^,^75^ on RD (12 μM) equilibrated in buffer A plus 2 mM CaCl_2_. EDTA was added at time t = 0 to a final concentration of 5 mM (i.e. residual free calcium below 20 nM), and after 10 sec of mixing, QELS data were similarly collected.

### Limited Proteolysis of CyaA by trypsin followed by mass spectrometry

Samples of hCyaAm in buffer A in the presence of 2 or 0.5 mM CaCl_2_ were incubated at 20 °C with trypsin (T8802, Sigma). Final protein concentrations of CyaA and trypsin were 800 and 20 nM, respectively. After 30, 90, 180, 420 min, 24 and 30 h (2 mM CaCl_2_) and 2, 5, 10, 20, 30, 90, 180, 330 min, 24, 29 & 48 h (0.5 mM CaCl_2_) of incubation, the proteolysis was stopped by adding the trypsin inhibitor AEBSF (4-(2-Aminoethyl) benzenesulfonyl fluoride hydrochloride) at a final concentration of 200 μM. The reaction was quenched further by immerging the sample tubes in liquid nitrogen. Samples were stored at −20 °C prior mass spectrometry analysis (MS). A cleaning step using C18 Ziptips^®^ was used for all samples and eluted in 10 μL (AcN/H2O/HCOOH) (75/5/0.1) (v/v/v). Peptides were dried down and resuspended in 20 μL solvent A (H_2_O: Acetonitrile: FA; 98:2:0.1) prior to MS analysis.

Digests were analysed on an LTQ-Orbitrap Velos instrument (Thermo Fisher Scientific, Bremen) equipped with a nano-HPLC Ultimate 3000 system (Dionex, Amsterdam, The Netherlands). Five microliters of each sample were loaded on a C18 trap column (300 μm inner diameter × 5 mm; Dionex) and peptides were further separated on an in-house packed 15 cm nano-HPLC column (75 μm inner diameter) with C18 resin (3 μm particles, 10 nm pore size, Reprosil-Pur Basic C18-HD resin, Dr. Maisch GmbH, Ammerbuch-Entringen, Germany). Sample loading was performed with a flow rate of 30 μL/min for 5 min followed by a flow rate of 300 nL/min for peptide separation on the analytical column. A 40 min gradient was used with the following conditions: 5 min 4% solvent B (H_2_O: Acetonitrile: FA; 20:80:0.08), 4–40% solvent B for 15 min, 40–95% solvent B for 0.1 min, 95% solvent B for 5 min, and 15 min 4% solvent B for column equilibration. The instrument was used in data dependent acquisition mode. After a survey scan in the Orbitrap (resolution 60,000), the 15 most intense precursor ions were selected for CID fragmentation in the ion trap. The normalized collision energy was set to 35 eV for 10 ms. Minimum signal threshold for triggering an MS/MS event was set to 5,000 counts. For internal mass calibration, the *m/z* 455,120025 ion was used as a lock mass. Charge state screening was enabled, and precursors with unknown charge state or a charge state of 1 were excluded. Dynamic exclusion was enabled for 90 s.

Raw files were processed with Mascot v.2.4.1 as search engine on Proteome Discoverer version 1.4.0.288 (Thermo Fisher Scientific, Bremen) against a homemade database. Trypsin was chosen as the specific enzyme, with a maximum number of two missed cleavages. Possible modifications included oxidation of methionines, which was set to variable. The mass tolerance for MS was set to 10 ppm and 0.5 Da was used for MS/MS.

### Thermal stability of hCyaAm

Temperature-induced denaturation of hCyaAm in the presence of various concentrations of CaCl_2_ was followed by intrinsic fluorescence of tryptophan on a JASCO FP 8200 spectrofluorimeter (Jasco, Tokyo, Japan) using a 1 cm path length quartz cell (111-QS, 10 × 10 mm, Hellma Analytics). CyaA samples were prepared in buffer A to reach a final concentration of 50 nM in the presence of various concentrations of CaCl_2_ (0, 0.2, 0.5, 1, 2 and 3 mM). The excitation wavelength was set at 290 nm, both emission and excitation slits were fixed at 5 nm and fluorescence emission spectra were recorded from 300 to 400 nm. Fluorescence data are reported as the ratio of fluorescence intensities at 320 and 360 nm (RFI 320/360). Melting curves were analysed using Kaleidagraph, Version 4.1.3, Synergy Software as already described^32^ to extract the melting temperature (Tm) values. A concentration of 50 nM of hCyaAm was used because temperature-scanning experiments followed by DLS (NanoZS, Malvern) indicated that above 200 nM the protein aggregates as temperature increases while below 100 nM the protein solution remains monodispersed on the timescale of the experiment.

### Permeabilization of large unilamellar vesicles

Permeabilization of large unilamellar vesicles (LUVs) was assayed by fluorescence recovery using co-encapsulated ANTS (fluorescent probe) and DPX (quencher) molecules^12^,^14^,^76^. LUV were prepared at a lipid concentration of 10 mM made of POPC:POPG:Cholesterol at a molar ratio of 3:1:1 in buffer A and containing 20 mM ANTS and 60 mM DPX. LUVs were done by reverse phase evaporation and sequential filtration through 0.8, 0.4 and 0.2 μm polycarbonate filters as previously described^12^,^77^. Non-trapped probes were removed by desalting the LUVs samples through a G-25 Sephadex column equilibrated with buffer A. The LUVs exhibited hydrodynamic diameters of 200 ± 20 nm, as checked by DLS on a NanoZS instrument (Malvern) as described elsewhere^31^,^75^. For permeabilization assays, the LUVs (0.5 mM lipid final concentration) were incubated at 25 °C under constant stirring. ANTS fluorescence was recorded continuously (Ex.: 360 nm, Em.: 515 nm, bandwidths 5 nm) to set the baseline. Then, a final concentration of CyaA (hCyaAm, M-CyaA or U-CyaA) at 50 nM was added and fluorescence was recorded for two hours.

### Haemolysis of erythrocytes

The hemolytic activity of CyaA was determined on sheep erythrocytes as previously described^20^. Sheep erythrocytes were washed several times with buffer A and resuspended at 5 × 10^8^ cells/mL. After the final wash, cells were incubated in buffer A in the presence of 2 mM CaCl_2_, 2 mM EDTA or buffer A alone. The hCyaAm and M-CyaA species were used as such, or desalted on G25 equilibrated in buffer A alone. To obtain the U-CyaA sample, CyaA in 6 M urea was desalted onto a G25 column equilibrated with buffer A and complemented with 2 mM calcium. For the experiments in the absence of free calcium in solution, the U-CyaA sample was once again desalted in buffer A to remove any free calcium in solution. Any remaining calcium corresponded to calcium ions bound to CyaA. The hemolytic activity was measured after an overnight incubation at 37 °C by quantifying the amount of hemoglobin released at 540 nm (and of intracellular content at 405 nm). Complete lysis was obtained by addition of 0.1% Triton X100.

### CyaA translocation and cAMP production in erythrocytes

The invasive activity of CyaA was determined by measuring the intracellular cAMP accumulation. The erythrocytes were incubated with different concentrations of CyaA at 37 °C for 20 min, then cells were chilled on ice, centrifuged at 4 °C at 2 500 rpm for 5 min, and resuspended in buffer A supplemented with 4 mM EDTA. The cells were centrifuged equivalently and the pellets were resuspended in 200 μL of buffer A complemented with 4 mM EDTA. After transfer into new tubes, the samples were lysed with 400 μL of 0.1 N HCl, and boiled for 5 min at 100 °C (to inactivate any remaining adenylate cyclase). The solutions were neutralized by addition of 400 μL of 0.1 N NaOH. The insoluble material was removed by centrifugation at 14 000 rpm for 10 min. The soluble fraction containing cAMP was stored at −20 °C and the intracellular cAMP content was determined by a competitive ELISA immunoassay^56^. A 96 well ELISA plate was coated with cAMP-BSA in 0.1 M Na_2_CO_3_, pH 9.5 (dilution 1:3000) (50 *μ*L/well) and incubated overnight at 4 °C. The next day, all wells were washed with buffer A complemented with 0.1% Tween 20. The wells were then saturated with buffer A complemented with 0.1% Tween 20, 1% BSA and incubated for 90 min at 30 °C under constant agitation. After incubation, wells were washed further with buffer A complemented with 0.1% Tween 20. Cyclic AMP samples where diluted twice with 40 mM Hepes, 300 mM NaCl, 0.2% Tween 20 and 100 *μ*L was distributed into each well. A standard curve of cAMP was prepared from the same plate as the samples. Anti-sera cAMP 1818-II (Homemade antibody from rabbit) was diluted into buffer A complemented with 0.1% Tween 20, 1% BSA and 50 *μ*L of this solution was added to each well. The plate was incubated for 3 hours at 30 °C under agitation. After incubation, the wells were washed three times with buffer A complemented with 0.1% Tween 20. Afterwards, 100 *μ*L of anti-rabbit IgG alkaline phosphatase (Sigma, Life Science) diluted into buffer A complemented with 0.1% Tween 20, 1% BSA was distributed to each well. The plates were incubated for 1 h at 30 °C under agitation. After incubation, the wells were washed four times with buffer A complemented with 0.1% Tween 20, followed by incubation for at least 1 hour at 30 °C with 100 *μ*L 4-Nitrophenyl phosphate disodium salt hexahydrate/well (PA substrate) (Sigma, Life Science) diluted in 50 mM Tris, 5 mM MgCl, pH 9.5. Absorbance values were read at 405 nm with a TECAN micro plate reader.

### Small angle X-ray scattering

SAXS measurements were carried out on the SWING beamline of the SOLEIL Synchrotron Radiation Facility (Saint-Aubin, France). The sample to detector (Aviex CCD) distance was set to 1988 mm, allowing reliable data collection over the momentum transfer range 0.06 nm^−1^ < q < 5 nm^−1^ with q = 4πsinθ/λ where 2θ is the scattering angle and λ the wavelength of the *X*-rays (λ = 0.1 nm). To separate the monomers from multimers, SAXS data was collected on solutions eluting from the on-line Size-Exclusion High-Performance Liquid Chromatography column (SE-HPLC Bio-SEC3, Agilent) available at SWING and directly connected to the SAXS measuring cell. The sample (monomeric hCyaAm at a concentration of 2.7 g/L, *i.e.*, 15 μM) was injected onto the column equilibrated with buffer A with 4 mM CaCl_2_ added. The protein was eluted at a flow rate of 200 μL/min. Temperature of the SAXS measurements in the SAXS capillary and in the column during elution was 15 °C. During elution scattering patterns (frames) were recorded for 1.5 s with a dead time between frames of 0.75 s. For each frame, the protein concentration (about 0.5 mg*/*mL at the top of elution peak) was estimated from UV absorption at 280 nm using a spectrometer located upstream of the SAXS measuring cell. The elution profile shown in Fig. 8A revealed a very small amount of multimers clearly separated from the monomers. Twenty identical frames corresponding to the main elution peak were averaged. A large number of frames were collected before the void volume and averaged to account for buffer scattering. SAXS data was normalized to the intensity of the incident beam and background (*i.e.*, the elution buffer) subtracted using the programs FoxTrot (courtesy of SWING beamline) and Primus^78^. The scattered intensities were plotted on an absolute scale using water scattering.

The shape of CyaA was determined by using the *ab initio* modelling program DAMMIN that describes the protein as an ensemble of elementary beads (dummy atoms) arranged on a hexagonal compact lattice whose scattering pattern is identical to the experimental curve within experimental uncertainty^60^. One hundred runs yielded as many models that were superimposed and compared using the DAMAVER suite^61^. The superposition is performed by minimizing the Normalized Spatial Discrepancy (NSD) in the Supcomb routine^79^ between pairs of models. A value smaller than 1 indicates that the two compared shapes are similar. For each model, its NSD is determined with respect to all other 99 models and an average NSD value is computed. All models can then be ranked by increasing average NSD. Supcomb was also used to superimpose pseudo atomic models of RD over dummy atom models of CyaA.

### Size exclusion Chromatography followed by a Tetra Detector Array (SEC-TDA)

Analytical size exclusion chromatography was performed on a Superdex200 10/300 column (GE Healthcare Life Sciences) and was controlled by a GPCmax module connected on-line to a tetra detector array (TDA) model 302 (Malvern Instruments Ltd). The oven of the TDA contained (i) a static light scattering cell with two photodiode detectors, at 7° for low angle (LALS) and at 90° for right angle laser light scattering (RALS), (ii) a deflection refractometer, (iii) a photometer and (iv) a differential viscometer. Protein concentration was determined using both the photometer and the deflection refractometer. The RALS data coupled to the concentration provided the molecular mass. The SEC is also coupled on-line to a quasi-elastic dynamic light scattering detector (μV, Malvern Instruments Ltd), which provides the hydrodynamic radius of eluting species. All solutions were filtered on 0.2 μm filters and allowed to equilibrate at 20 °C before SEC experiments and sample analyses were performed at 25 °C in the oven of the TDA. All experimental sequences comprised calibration injections of BSA and PEO used for TDA calibration (200 μL at 2 mg/mL). All data were acquired with Omnisec software (Malvern Instruments Ltd) and processed following already described procedures^28^,^30^,^33^,^57^. Estimation of shape and time-averaged apparent hydration were done as previously described^57^.

## Additional Information

**How to cite this article:** Cannella, S. E. *et al*. Stability, structural and functional properties of a monomeric, calcium–loaded adenylate cyclase toxin, CyaA, from *Bordetella pertussis. Sci. Rep.* **7**, 42065; doi: 10.1038/srep42065 (2017).

**Publisher's note:** Springer Nature remains neutral with regard to jurisdictional claims in published maps and institutional affiliations.

## Supplementary Material

Supplementary Information
