# Supplementary material for: Stability, structural and functional properties of a monomeric, calcium–loaded adenylate cyclase toxin, CyaA, from Bordetella pertussis
Source: Sci Rep. 2017 Feb 10;7:42065. doi: 10.1038/srep42065 (PMC5301233; doi:10.1038/srep42065)
Supplement: Supplementary Information [file srep42065-s1.pdf]

Supplementary information to:

Stability, structural and functional properties of a monomeric, calcium–loaded adenylate cyclase toxin, CyaA, from *Bordetella pertussis*

Sara E Cannella[1], Véronique Yvette Ntsogo Enguéné[1], Marilyne Davi[1], Christian Malosse[1], Ana Cristina Sotomayor Pérez[1], Julia Chamot-Rooke[1], Patrice Vachette[2], Dominique Durand[2], Daniel Ladant[1*] and Alexandre Chenal[1*]

From [1] Institut Pasteur, UMR CNRS 3528, Chemistry and Structural Biology Department, 75724 PARIS cedex 15, France; [2] Institut de Biologie Intégrative de la Cellule, UMR 9198, Université Paris-Sud, F-91405 ORSAY Cedex, France

Supplementary Figures

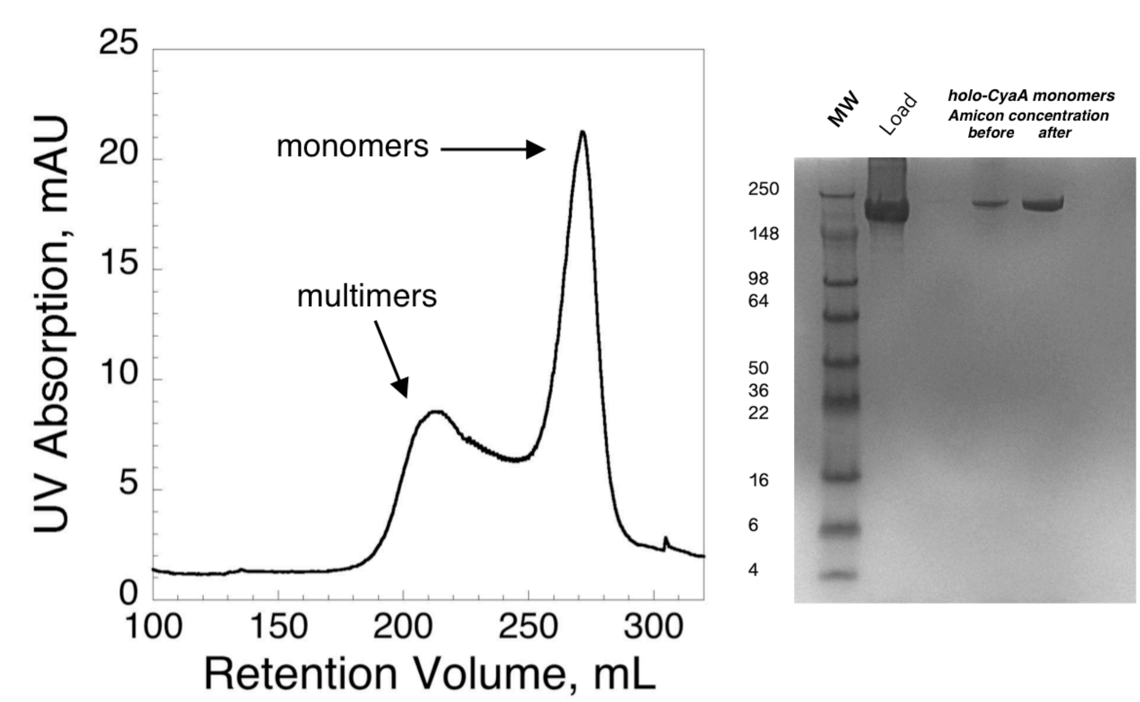

Supplementary Figure S1

Supplementary Figure S1: Left Panel: Refolding of CyaA into a monomeric toxin*:* the protein is refolded by Size Exclusion Chromatography, using a HiLoad 16/600 Superdex 200 pg column (GE Healthcare), as described in Karst *et al.*, 2014. Briefly, the Superdex 200 pg was equilibrated with 20 mM Hepes, 150 mM NaCl, 2 mM $CaCl_2$, pH 7.4. The sample consists of 5 mL of CyaA at 5 µM stored in 8 mM urea, 20 mM Hepes, pH 7.4. After elution, monomers and multimers were pooled separately. Proteins were then concentrated on Amicon and their hydrodynamic radius measured. Proteins were aliquoted and stored at -20°C. Right Panel: SDS PAGE of CyaA in urea before refolding, followed by samples of monomers of holo-CyaA before and after concentration on Amicon devices.

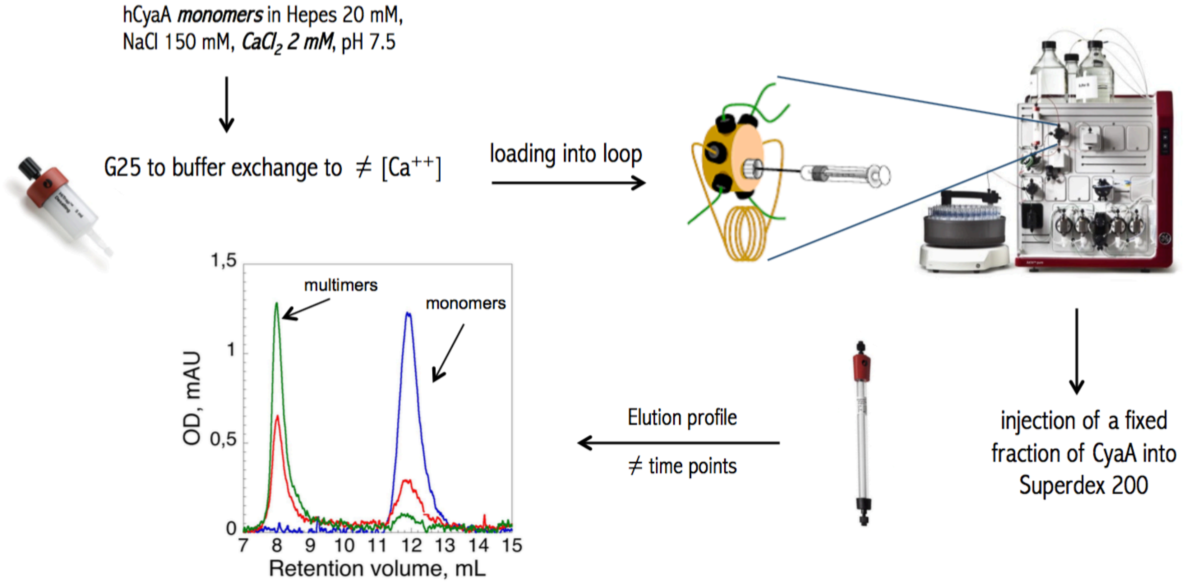

Supplementary Figure S2

Supplementary Figure S2: Scheme of the experimental setup to investigate the stability of CyaA over time. CyaA monomers in 20 mM Hepes, 150 mM NaCl, 2 mM $CaCl_2$, pH 7.4 were buffer exchanged on a G25 column in the presence of different concentrations of calcium and then concentrated on Amicon up to 1 µM of protein. One mL of CyaA (1 µM) was loaded into a loop of a Äkta Pure Chromatography System. At selected time points, an aliquot of 100 µL of CyaA was injected into a Superdex 200 10/300. Elution profiles of CyaA at different times and calcium concentrations were recorded and compared. The proportions of each species (monomers and multimers) were calculated by integrating the intensity of each peak.

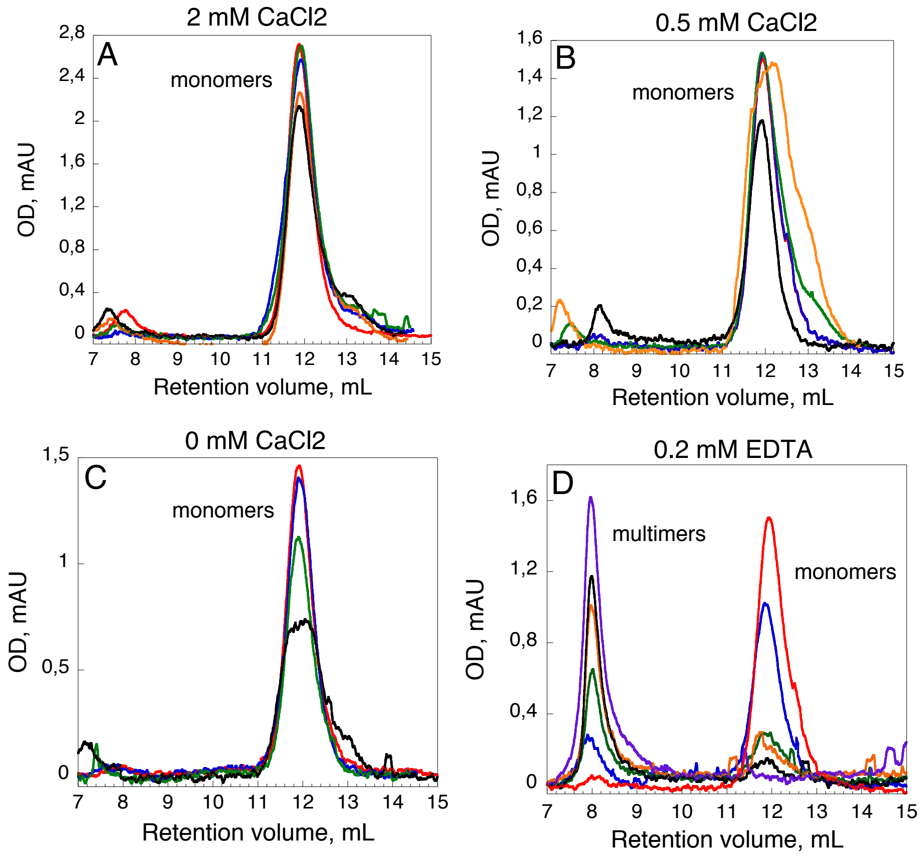

Supplementary Figure S3

Supplementary Figure S3. **Calcium-dependent stability of hCyaAm over time.** Chromatograms of hCyaAm samples at various time points. Panel A (buffer A complemented with 2 mM CaCl2), B (buffer A complemented with 0.5 mM CaCl2), panel C (buffer A) and panel D (buffer A complemented with 0.2 mM EDTA). Samples of hCyaAm at 1 μM were loaded into the injection loop of an Äkta Pure Chromatography System. At various time points, aliquots of CyaA were injected into a Superdex 200 10/300 column equilibrated with the same buffer as the hCyaAm sample loaded into the injection loop. The species of CyaA are defined according to their retention volumes, *i.e.,* multimers (8-9 mL) and monomers (11-13 mL). Panels A, B and C: Time zero: red, 12 hours: blue, 24 hours: green, 48 hours: orange, 72 hours: black; panel D: time zero: red, 1 hour: blue, 2 hours: green, 3 hours: orange, 4 hours: black, 5 hours: violet. Buffer A: 20 mM Hepes, 150 mM NaCl, pH 7.4.

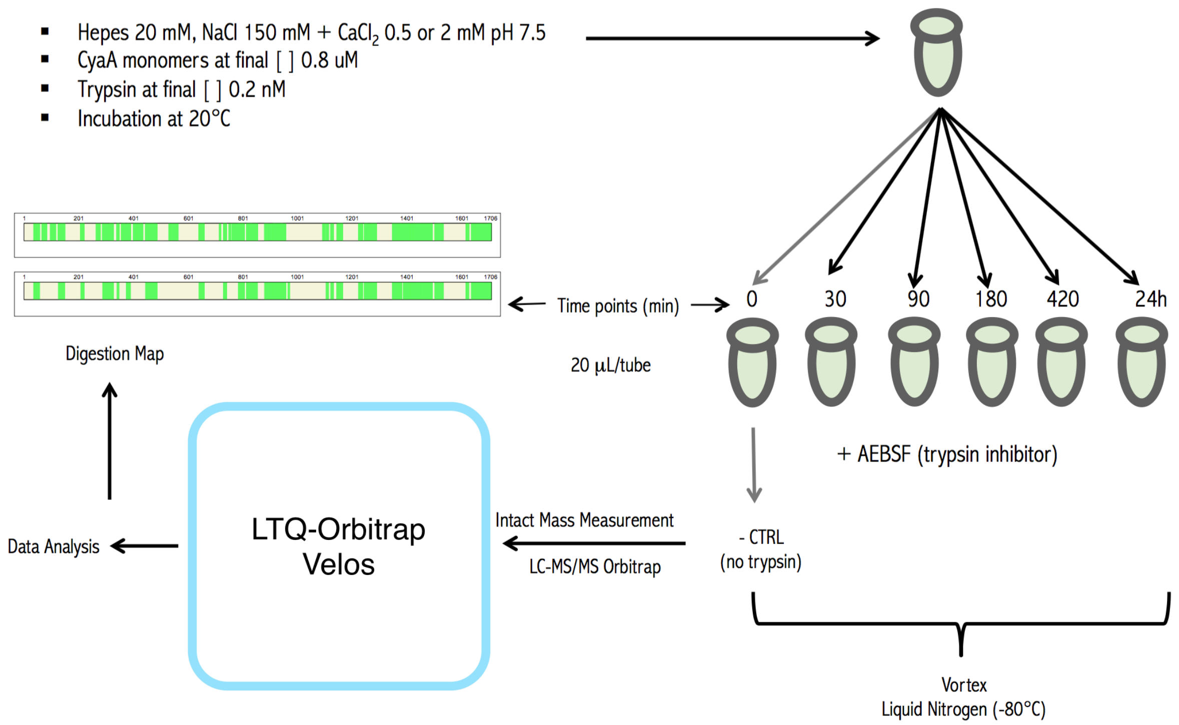

Supplementary Figure S4

Supplementary Figure S4: Scheme of the limited proteolysis of hCyaAm followed by Mass Spectrometry. CyaA monomers (0.8 µM) in 20 mM Hepes, 150 mM NaCl and either 0.5 or 2 mM $CaCl_2$ were incubated at 20°C with 20 nM trypsin. At various time points, (*i.e.* 30, 90, 180, 420 min and 24 h) the proteolytic reactions were stopped by adding AEBSF at 200 µM and aliquots were frozen into liquid nitrogen. Digests were analyzed by mass spectrometry on an LTQ-Orbitrap Velos instrument (Thermo Fisher Scientific). Five microliters of each sample were loaded on a C18 trap column and peptides were further separated on an in-house packed nano-HPLC column. Data were processed with Mascot v.2.4.1 as search engine on Proteome Discoverer v.1.4.0.288 against a homemade database.

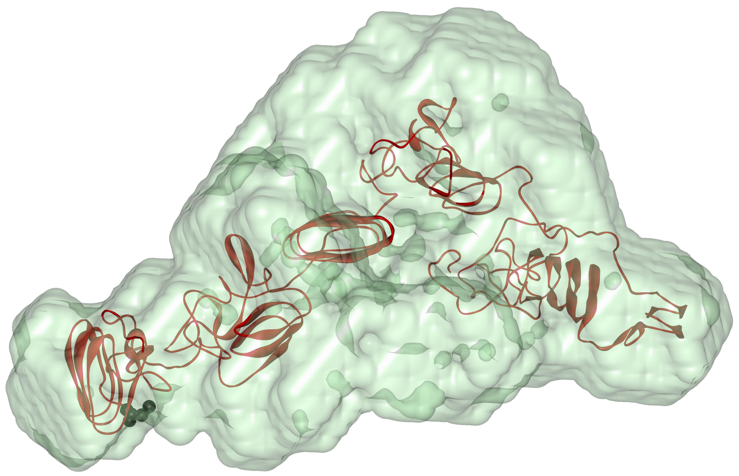

Supplementary Figure S5

Supplementary Figure S5: Most typical DAMMIN model of CyaA with the pseudo-atomic model of RD (red cartoon representation) superimposed. The black spheres indicate the N-terminal residues of RD.

Supplementary Table S1: Proteolytic sites of hCyaAm in the presence of 0.5 mM calcium identified by mass spectrometry

CyaA native numbering of proteolysis by trypsin	CyaA full length, 20 mM Hepes, 150 mM NaCl, 0.5 mM $CaCl_2$ first time point of occurrence of proteolysis at each amino acid position						
	**0 min**	**2 min**	**30 min**	**90 min**	**180 min**	**420 min**	**24 hours**
R15			X				
K34			X				
R41	X						
K58			X				
K65			X				
R88					X		
R95	X						
R124	X						
R158					X		
K175			X				
R206	X						
R224							X
R237	X						
R262	X						
R265						X	
R285	X						
K312		X					
R338		X					
K355			X				
K374			X				
R399	X						
R435	X						
R443	X						
R527			X				
R711					X		
K728			X				
R746					X		
K758	X						
R812	X						
R832	X						
R836			X				
R879			X				
K891	X						

K908	X						
K962				X			
R984							X
R1101			X				
K1118			X				
R1144	X						
R1220	X						
K1242			X				
R1344	X						
K1383				X			
K1410		X					
R1481		X					
K1498			X				
K1506		X					
R1612		X					
R1632			X				
R1652							X
K1670		X					
R1691					X		

Supplementary Table S2: Proteolytic sites of hCyaAm in the presence of 2 mM calcium identified by mass spectrometry

	**CyaA full length, 20 mM Hepes, 150 mM NaCl, 2 mM $CaCl_2$ first time point of occurrence of proteolysis at each amino acid position**						
CyaA native numbering of proteolysis by trypsin	**0 min**	**2 min**	**30 min**	**90 min**	**180 min**	**420 min**	**24 hours**
R15					X		
K32						X	
K34				X			
K65					X		
R88					X		
R158							X
R165							X
R206			X				
R235					X		
R237			X				
R262							X
R285					X		
K331							X
R338			X				
K355					X		
R399			X				
R443				X			
R461			X				
K638						X	
R711					X		
K720						X	
R727					X		
R746					X		
K782					X		
K798			X				
R832					X		
R877				X			
K908					X		
R957						X	
K962							X
R984							X
K1088					X		

R1101				X			
K1113						X	
R1220						X	
R1241					X		
R1255				X			
R1338							X
R1344					X		
R1367						X	
R1409					X		
R1481			X				
K1498					X		
R1612					X		
R1632							X
R1692						X	

Supplementary Table S3: Proteolytic sites of RD in the presence of 0.5 mM calcium identified by mass spectrometry

	**RD, 20 mM Hepes, 150 mM NaCl, 0.5 mM $CaCl_2$ first time point of occurrence of proteolysis at each amino acid position**						
CyaA native numbering of proteolysis by trypsin	**0 min**	**5 min**	**30 min**	**90 min**	**180 min**	**360 min**	**24 hours**
R1101		X					
K1118			X				
R1220		X					
R1255		X					
K1266		X					
R1367		X					
R1409	X						
R1452						X	
R1454		X					
K1506		X					
R1612		X					
R1632		X					
R1652		X					

Supplementary Table S4: Proteolytic sites of RD in the presence of 2 mM calcium identified by mass spectrometry

CyaA native numbering of proteolysis by trypsin	RD, 20 mM Hepes, 150 mM NaCl, 2 mM $CaCl_2$ first time point of occurrence of proteolysis at each amino acid position						
	**0 min**	**5 min**	**30 min**	**90 min**	**180 min**	**360 min**	**24 hours**
R1101		X					
K1118	X						
R1220		X					
R1255		X					
K1266						X	
R1367	X						
R1409	X						
R1452					X		
R1454		X					
R1481	X						
K1506		X					
R1612	X						
R1632		X					
R1652		X					

Supplementary Table S5: Comparison of the hydrodynamic parameters obtained by SEC-TDA-μV, AUC and SAXS experiments. SEC-TDA-μV and AUC experiments were performed at 20°C; SAXS at 15°C. The viscosity and density of the buffer are 1.03 cP and 1.006 g/mL at 20°C, respectively. The partial specific volume is computed using Sednterp.

	SEC-TDA-μV	AUC	SAXS	DAMMIN models[a]	Computed data
MM, kDa	175 ± 10	176 ± 5	180 [b]<br>169 [c]	-	177.5
$\bar{v}$, mL/g	-	0.725 ± 0.005			0.725
[η], mL/g	5.5 ± 0.3	-		5.5 ± 0.2	-
D_t,$10^{-7}$$cm^2$/s	3.9 ± 0.4	-		4.0 ± 0.1	-
S, 10^{-13} sec	-	7.3 ± 0.1		8.2 ± 0.1	-
R_H, nm	5.3 ± 0.4	5.6 ± 0.2		5.3 ± 0.1	-
R_O, nm	-	-		-	3.70
f/f_0	1.43 ± 0.1	1.51 ± 0.1		1.44± 0.02	-
D_{max}, nm			16.0 ± 1	17.0 ± 0.5	
R_g, nm			4.47±0.02[d]<br>4.53±0.02[e]	4.55 ± 0.06	
R_g/R_H	0.86	0.81			
ν	2.6	-			-
δ, g.g^{-1}	1.4	-			-
a/b (prolate)	1.39	-			-
a, nm	6.8	-			-
b, nm	4.9	-			-
a/b (oblate)	1.41	-			-
a, nm	6.2	-			-
b, nm	4.4	-			-

[a] The parameters given below were obtained from the ten most typical DAMMIN models using the hydrodynamic modelling program HYDROPRO [1]. Similar values were obtained using the program SOMO [2].
[b] Value of the molecular mass derived from I(q) using the program SAXSMoW
[c] Value of the molecular mass derived from I(q) using the program Scatter
[d] R_g obtained with the Guinier approximation in the range $0.4<qR_g<1$
[e] R_g obtained from the P(r) function

List of parameters - abbreviations

MM, molecular mass, kDa
$[\eta]$, intrinsic viscosity, mL/g
ν, viscosity increment, Simha-Saito shape factor
δ, hydration, $g_{H_2O}.g^{-1}_{protein}$, gram of water per gram of protein
f/f_0, frictional ratio of the protein
f, frictional coefficient of the protein, $g.cm.s^{-2}$
f_0, frictional coefficient of an anhydrous sphere of the mass of the protein, $g.cm.s^{-2}$
R_H, hydrodynamic radius of the protein, nm
R_0, radius of an anhydrous sphere of the mass of the protein, nm
R_g, radius of gyration, nm
R_g/R_h
D_t, translational diffusion coefficient, 10^{-7} cm^2/s
D_{max}, nm
S, sedimentation coefficient, Svedberg, 10^{-13} sec
$\bar{\nu}$, partial specific volume, mL/g

Supplementary References

1 Ortega, A., Amoros, D. & Garcia de la Torre, J. Prediction of hydrodynamic and other solution properties of rigid proteins from atomic- and residue-level models. *Biophys J* **101**, 892-898, doi:10.1016/j.bpj.2011.06.046 (2011).

2 Brookes, E., Demeler, B., Rosano, C. & Rocco, M. The implementation of SOMO (SOlution MOdeller) in the UltraScan analytical ultracentrifugation data analysis suite: enhanced capabilities allow the reliable hydrodynamic modeling of virtually any kind of biomacromolecule. *Eur Biophys J* **39**, 423-435, doi:10.1007/s00249-009-0418-0 (2010).
